# Personalized prediction of early childhood asthma persistence: A machine learning approach

**DOI:** 10.1371/journal.pone.0247784

**Published:** 2021-03-01

**Authors:** Saurav Bose, Chén C. Kenyon, Aaron J. Masino

**Affiliations:** 1 Department of Biomedical and Health Informatics, Children’s Hospital of Philadelphia, Philadelphia, Pennsylvania, United States of America; 2 Center for Pediatric Clinical Effectiveness, Children’s Hospital of Philadelphia, Philadelphia, Pennsylvania, United States of America; 3 Department of Pediatrics, Perelman School of Medicine at the University of Pennsylvania, Philadelphia, Pennsylvania, United States of America; 4 Department of Anesthesiology and Critical Care, Perelman School of Medicine at the University of Pennsylvania, Philadelphia, Pennsylvania, United States of America; Washington University in St. Louis, UNITED STATES

## Abstract

Early childhood asthma diagnosis is common; however, many children diagnosed before age 5 experience symptom resolution and it remains difficult to identify individuals whose symptoms will persist. Our objective was to develop machine learning models to identify which individuals diagnosed with asthma before age 5 continue to experience asthma-related visits. We curated a retrospective dataset for 9,934 children derived from electronic health record (EHR) data. We trained five machine learning models to differentiate individuals without subsequent asthma-related visits (transient diagnosis) from those with asthma-related visits between ages 5 and 10 (persistent diagnosis) given clinical information up to age 5 years. Based on average NPV-Specificity area (ANSA), all models performed significantly better than random chance, with XGBoost obtaining the best performance (0.43 mean ANSA). Feature importance analysis indicated age of last asthma diagnosis under 5 years, total number of asthma related visits, self-identified black race, allergic rhinitis, and eczema as important features. Although our models appear to perform well, a lack of prior models utilizing a large number of features to predict individual persistence makes direct comparison infeasible. However, feature importance analysis indicates our models are consistent with prior research indicating diagnosis age and prior health service utilization as important predictors of persistent asthma. We therefore find that machine learning models can predict which individuals will experience persistent asthma with good performance and may be useful to guide clinician and parental decisions regarding asthma counselling in early childhood.

## 1. Introduction

Asthma is a chronic inflammatory disease of the airways characterized by recurrent wheezing that affects 7.1 million American children [1]. While early diagnosis of asthma may be beneficial for those with treatment responsive phenotypes, there is a large population of children that receive an incident asthma diagnosis who ultimately do not experience chronic (or persistent) asthma symptoms [2]. In such cases, early diagnosis may lead to unnecessary treatment, potential associated side-effects, and alterations in quality of life for both children and their families [3]. As such, upon initial diagnosis of early childhood asthma, it is important to parents and clinicians to have an accurate prognosis as to whether asthma will persist as a chronic condition. The ability to better distinguish, on an individual basis, children likely to experience persistent diagnosis from those with a transient diagnosis would, therefore, be valuable.

Prior research of asthma-related predictive models is primarily focused on incidence and progression. Asthma incidence prediction models provide risk estimates for a future asthma diagnosis in individuals without a prior diagnosis. Early models [4–6] were rule based systems based on the occurrence of early childhood wheezing episodes. More recently, statistical models [1,3] have been developed that identify pre-school children with asthma-like symptoms who are at high risk of future asthma diagnosis. Asthma progression models predict exacerbation of symptoms and physiological characteristics that lead to adverse outcomes such as emergency department (ED) visits. Researchers have used electronic questionnaire responses, patient telemonitoring data [7,8], and administrative data in conjunction with patient attributes and environmental variables [9,10] to develop progression prediction models.

Asthma persistence refers to continuation of symptoms, with or without exacerbations. Progression implies persistence, however the converse is not necessarily true [2]. Research on asthma persistence prediction models is, to our knowledge, limited to a 2007 birth cohort study [11]. In that study, the authors estimated univariate correlations of covariates with asthma persistence and covariate-adjusted risks of persistence, respectively. Both analyses yielded population-level estimates that found hospitalization to be an important determinant of asthma persistence.

None of the aforementioned studies presented models expressly designed to provide individualized prediction of asthma persistence. In this study, we developed and analyzed multiple machine learning models designed to predict individual asthma persistence. Specifically, given clinical input for a child under the age of 5 years with an incident asthma diagnosis, our models predict whether subsequent asthma diagnosis will occur by age 10 years. We trained and evaluated models on electronic health record (EHR) data for 9,934 children. We show that the models are able to distinguish between individuals who will experience persistent asthma and those who will not with good performance. We additionally provide an examination of the important model input features to ensure clinical relevance and plausibility. To the best of our knowledge, ours is the first study to describe a comprehensive investigation of modern machine learning algorithms for persistent asthma diagnosis prediction in children using large-scale EHR data.

## 2. Materials and methods

### 2.1. Study setting

We implemented a retrospective, cohort study using data derived from the Pediatric Big Data (PBD) resource at the Children’s Hospital of Philadelphia (CHOP) (a pediatric tertiary academic medical center). The PBD resource includes data collected from the CHOP Care Network (a primary care network of over 30 sites), and CHOP Specialty Care and Surgical Centers. The PBD resource contains demographic, encounter, medication, procedure, and measurement (e.g. vital signs, laboratory results) elements for a large, unselected population of children who utilize the CHOP healthcare system. All data in the PBD resource were extracted from the CHOP EHR by non-study staff personnel. All PHI identifiers except for encounter dates were removed from the dataset prior to transfer to the study database. The Institutional Review Board at the Children’s Hospital of Philadelphia approved this research study and waived the requirement for consent.

### 2.2. Inclusion criteria

**Fig 1:**
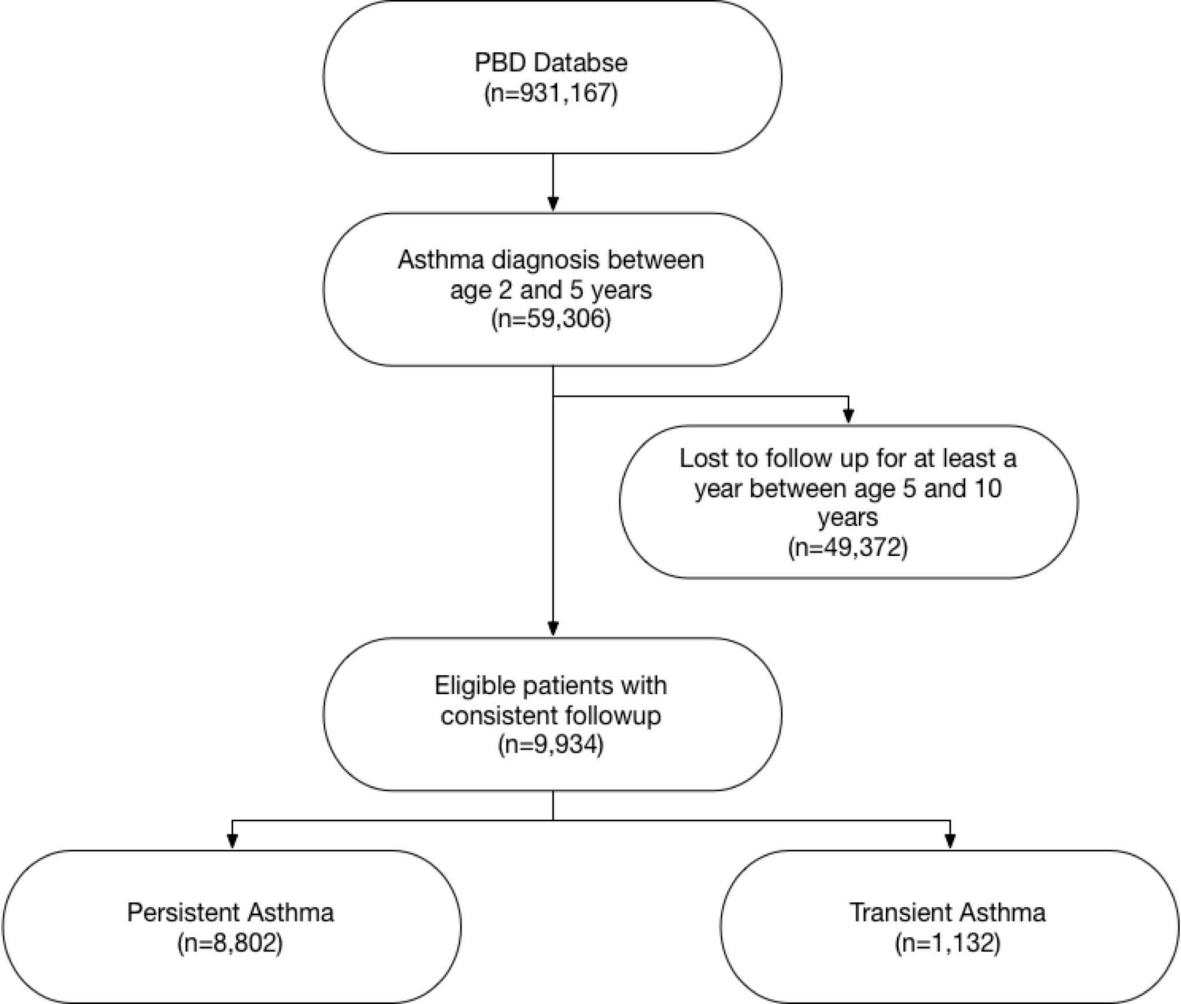
Study flow diagram. Excluded patients that were lost to follow up between age of 5 and 10 years.

The study cohort included children with an incident asthma diagnosis between the ages of 2 and 5 years, recorded during a face-to-face healthcare encounter (inpatient stay, ambulatory visit, or emergency department visit) between January 1, 2005 and December 31, 2016. We defined an *asthma diagnosis* as the presence in the medical record of any sub-code of the International Classification of Diseases, Ninth Revision (ICD-9) code 493 or ICD-10 code j45. To ensure individuals were not lost to follow-up, individuals must also have had at least one healthcare visit with a recorded ICD diagnosis (not necessarily asthma related) every year post 5 years of age up until 11 years of age. Our inclusion criterion yielded a dataset with 9,934 children (See Fig 1).

For the purpose of this study, we considered an individual to have *persistent asthma* if **all of the following conditions are satisfied**:

1. Initial asthma diagnosis occurred between ages 2 and 5 years (based on presence of an asthma ICD9/10 code as described above)
2. At least one additional asthma diagnosis occurred between ages 5 and 10 years
3. An asthma-related medication (see S1 File) was prescribed at least once at a visit that (a) coincided with or followed the first asthma diagnosis visit, and (b) occurred after the age of 2 years.

Our cohort contains two groups: (1) *children with persistent asthma diagnosis* (n = 8802), i.e. those who satisfied the condition for persistence; and (2) *children with transient asthma diagnosis* (n = 1132), i.e. those with an initial diagnosis prior to age 5 years who did not satisfy the condition for persistence. This resulted in an analysis dataset with approximately 89% positive instances and 11% negative instances (see Table 1). Our rationale for developing predictive models for asthma diagnosis over the age of 5 years was guided by the National Heart Lung Blood Institute’s Expert Report Panel’s asthma guidelines [12] which divides childhood asthma diagnosis and management recommendations into three age groups 0–4, 5–11, and 12–17. Although asthma diagnosis in children aged 0–4 years may be appropriate, it is controversial, as, many children who don’t have classic asthma (recurrent chronic airway obstruction independent of viral triggers) wheeze in the setting of viral illnesses [13] and are diagnosed with asthma. From an epidemiological perspective, this “recurrent viral-associated wheezing” is far less common in children aged 5 years and older and the diagnosis of asthma becomes more reliable.

**Table 1:** Study cohort demographics at the time of first asthma diagnosis.

Category	Variable Name	Positive	Negative
Clinical			
	Number of children	8802	1132
	Age (years)	Median (3.07)	Median (3.17)
		Range (0.15, 4.99)	Range (0.29, 4.99)
	Number of asthma related visits	Median (5)	Median (2)
		Range (1, 179)	Range (1, 25)
	Number of non-asthma visits	Median (24)	Median (25)
		Range (0, 1360)	Range (0, 394)
Race (Percent)			
	White	46% [4077]	72% [818]
	Black	44% [3921]	17% [191]
	Unknown	6% [534]	8% [88]
	Asian	2% [195]	2% [26]
	Multiple	1% [69]	<1% [7]
	American Indian/ Alaska Native	<1% [5]	<1% [2]
	Native Hawaiian/ Other Pacific Islander	<1% [1]	0% [0]
Gender (Percent)			
	Male	60% [5282]	54% [611]
	Female	40% [3520]	46% [521]
Ethnicity (Percent)			
	Non-Hispanic	93% [8208]	93% [1058]
	Hispanic	6% [529]	6% [65]
	Unknown/No information	1% [65]	1% [9]

Percentages are relative to positive and negative groups. Values in brackets indicate number of individuals.

### 2.3. Feature selection

We considered 648 features (4 numerical, 644 categorical) including demographics, geographic location, care site information, insurance information, and clinical history. Demographic features include self-reported race, ethnicity, gender, and language spoken. Geographic location is characterized by the patient state of residency at the time of the first asthma diagnosis. Care site information features include place of service (e.g. office visit, emergency room), care site specialties, and provider specialties at the time of first asthma diagnosis. Insurance information features include a binary variable indicating Medicaid enrollment at any time before or during the first visit with an asthma diagnosis. Finally, clinical history features include patient age (in years) at first and last asthma diagnoses prior to age 5 years, number of visits with an asthma related ICD9 or ICD 10 code, number of visits with a non-asthma related ICD9 or ICD10 code, and binary variables encoding the presence of a condition described by their EDC (Expanded Diagnostic Clusters) codes from the Adjusted Clinical Group (ACG) System [14,15] recorded during a visit. Additionally, within the clinical history features, we included indicator (yes/no) variables for procedures, medications, and measurements recorded in the EHR up to age 5 years. We used indicator variables (e.g. presence of a heart rate measurement) rather than numerical values (e.g. measured heart rate) because data may not be missing completely at random which precludes the use of imputation. We included 30 procedures for which at least 5% of the study cohort had the procedure at least once prior to age 5 years, all medications as identified by their Anatomical Therapeutic Chemical (ATC) Classification [16] codes prescribed to the patient prior to age 5 years, and lab measurements deemed plausibly predictive of persistent asthma diagnosis by our physician expert collaborators (see S2 and S3 Files).

To address potential model over-fitting due to noisy or correlated features, we evaluated filter and embedded feature selection methods [17,18] in our cross-validation training procedure. Among filter methods, we considered the chi square, ANOVA F-value and relief algorithms. For Chi Square and Anova F-value methods, we retained features where the univariate test statistic of association between the feature and the target label had a p-value ≤0.01. For the ReliefF and MultiSURF relief algorithms [19], we retained features with a positive feature importance score. Additionally, we considered embedded feature selection with our logistic regression, random forest and XGBoost models. We also considered the following novel combinations–(1) Chi Square followed by ReliefF, (2) Chi Square followed by MultiSURF, (3) Anova-F followed by Relief and (4) Anova-F followed by MultiSURF. Finally, we considered the filter methods and their combinations described above followed by embedded feature selection for the logistic regression, random forest and XGBoost models.

### 2.4. Class balance

Our study data consists of imbalanced data, in that a supermajority of individuals are in the persistent asthma group, which often degrades machine learning performance. To address this concern, we applied under sampling techniques that balance the training set (no change to the validation sets) and attempt to remove noisy instances from the training data. We specifically considered: (1) random under sampling, (2) edited nearest neighbors (ENN), (3) repeated edited nearest neighbors (R-ENN), (4) Tomek links. In random under sampling, class instances are balanced by randomly selecting a subsample of majority class instances equal in size to the minority class dataset. The resulting class balance with this method was 50/50. The ENN [20] method removes instances (of the majority class) whose class label differs from a majority of its k-nearest neighbors. We selected k = 5 in our study. The R-ENN method [21] repeats the ENN procedure until the majority of the k-nearest neighbors for every data point (of the majority class) have the same class label as the data point. Finally, a Tomek link is defined as a pair of instances which are each other’s nearest neighbor but are in different classes [22]. We identified Tomek links in the dataset and removed the corresponding majority class instances. The resultant class balance after employing either ENN, R-ENN or Tomek link removal cannot be predefined and depends on the initial class distribution and structure of the dataset. We also tried two combinations of under sampling techniques–(1) Tomek link removal followed by edited nearest neighbors and (2) Tomek link removal followed by repeated edited nearest neighbors. We also attempted to address class imbalance by modifying model training loss functions to weight instances inversely proportional to class frequencies.

### 2.5. Model training

**Fig 2:**
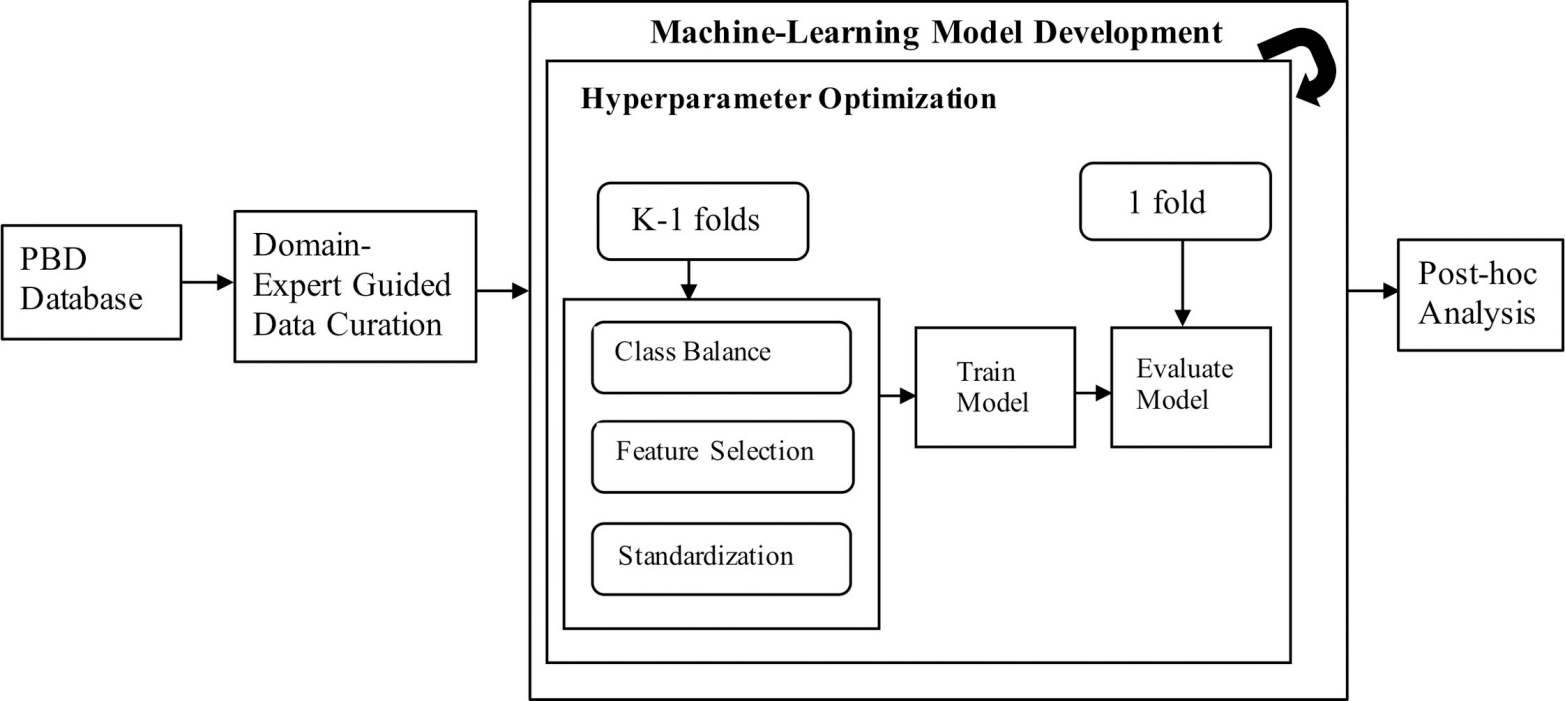
Experimental workflow. Data was obtained from the Pediatric Big Data (PBD) resource at CHOP. Physician domain expertise was used to identify the initial feature set. Model training and evaluation was performed using K-fold cross-validation (K = 5 in our study) twice to generate 10 unique validation folds and a corresponding set of training folds (K-1 folds). For each set of training folds, class balance, algorithmic feature selection and standardization of continuous variables was performed before training the model using particular hyperparameters. Thereafter, the trained model was evaluated on the corresponding validation fold. This process was repeated to scan the hyperparameter space using Bayesian optimization to find near optimal hyperparameters. Finally, inter-model performance was compared using statistical significance tests.

We trained five machine learning algorithms to discriminate between persistent and transient asthma diagnoses: naïve Bayes, logistic regression, k-nearest neighbors, random forest and gradient boosted trees (XGBoost). We selected these modeling methods to allow for varying model capacity ranging from linear to highly non-linear in order to address potential model under-fitting. The experimental workflow is outlined in Fig 2. The performance of each of these algorithms depends heavily on the choice of hyperparameters (i.e. model tuning parameters) [23]. As a step in the model training procedure, we used Bayesian optimization, also known as sequential-model-based optimization (SMBO) [24], to select near-optimal hyperparameters using the tree-structured Parzen estimator modeling strategy [25]. Hyperparameters can be used to modify model behavior including regularization terms which seek to control model over-fitting. Recent studies show that SMBO is more efficient at identifying near optimal hyperparameters than methods like grid search [26,27]. As required by the SMBO technique, we first defined a search space for each hyperparameter of a given model. This search space was then used by the SMBO algorithm to intelligently select hyperparameter combinations based on model performance. The hyperparameter search space for each of the machine learning algorithms is summarized in S1 Table.

**Fig 3:**
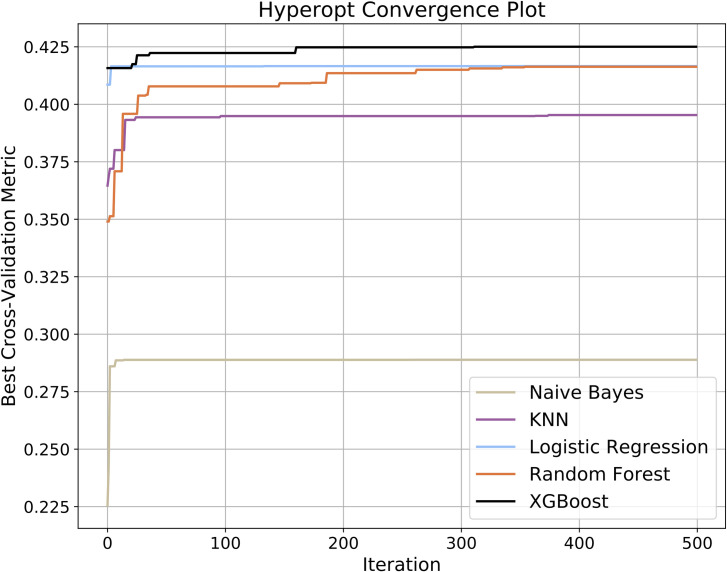
Hyperparameter convergence. HyperOpt iteratively chooses candidate hyperparameter combinations from a large hyperparameter space based on model performance on the prior iteration. The plot represents the highest performance up to a given iteration. For the ML algorithms in this study, there was no performance improvement after the 300^th^ iteration.

We define *P* as the set of all combinations of feature selection and class-balance techniques described in Section 2.3 and Section 2.4. For each element in *P*, we trained the machine learning models with a stratified cross validation (CV) approach that included the class balance and feature selection routines embedded in the CV procedure (see S1 Fig). First, we randomly divided the training set into K-folds (K = 5) in a stratified fashion. For each iteration, class balance and feature selection routines were performed on K-1 (training) folds. Numerical variables in the training folds were standardized to zero mean and unit standard deviation (with the exception of Naïve Bayes, for which feature standardization was not performed because the feature space must be non-negative values for Bernoulli and Multinomial Naïve Bayes classifiers.). Numerical variables in the k^th^ validation fold were standardized using the corresponding mean and standard deviation from the training folds. The machine learning algorithm was trained on the K-1 folds and evaluated on the k^th^ validation fold. The entire process was repeated twice resulting in a total of 10 unique validation folds. The hyperparameter optimization scheme uses the average of the evaluation metric for the 10 CV folds to select the next hyperparameter candidate values. We ran this hyperparameter tuning process for 2000 iterations. However, we found that hyperparameter selection converged in less than 300 iterations for the machine learning algorithms considered (see Fig 3).

### 2.6. Model evaluation

Receiver Operating Characteristic (ROC) curve analysis is commonly used to assess machine learning model performance, however studies have shown that in the presence of high class imbalance the *precision-recall* (PR) curve is more informative [28]. The area under the PR curve can be used as a point metric to summarize performance, however, numerical integration accuracy is generally poor when there are a limited number of precision-recall measurements. In such situations, *average precision* (AP) provides a more accurate performance assessment. As our study involved an imbalanced dataset where the negative class is the majority, we used the NPV-Specificity curve, where NPV indicates *negative predictive value*, instead of the PR curve. The NPV-Specificity curve is the equivalent of the PR curve when the negative class is the majority. We then used the area under the NPV-Specificity curve, denoted ANSA (Average NPV-Specificity Area), computed in an analogous manner to average precision as our evaluation metric. The null hypothesis of equal inter-model ANSA distributions was tested using Friedman’s Rank Sum test and post-hoc analysis of pair-wise comparison using the Exact Permutation test.

All of the models used in the study produce a numeric output which can be interpreted as the probability of experiencing persistent asthma. A positive or negative label is assigned by setting a threshold (0.5 by default) on the numeric output. We further compared model performance by setting a decision threshold for each model independently to yield a fixed specificity across all models and then computing standard point metrics: NPV, precision, sensitivity, and accuracy.

Finally, we performed a permutation feature importance analysis on our best performing model to better understand the model’s behavior. The feature importance is computed by measuring the change in the ANSA on the test set when the values in the dataset for a given feature are randomly shuffled among samples. Feature importance is reflected by a decrease in ANSA as compared to when the feature is not permuted, with higher importance indicated by a larger decrease.

We used Python’s imbalanced-learn [29] module to perform the class balancing routines, scikit-rebate [19] and scikit-learn’s [30] feature_selection module to perform feature selection, hyperopt [27] to tune the hyperparameters and ELI5 [31] to compute feature importance. XGBoost [32] was used to train the XGBoost model and scikit-learn’s implementation of ML models for all the other algorithms. All code is available at https://github.com/masino-lab/asthma-persistence-prediction.

## 3. Results

**Fig 4:**
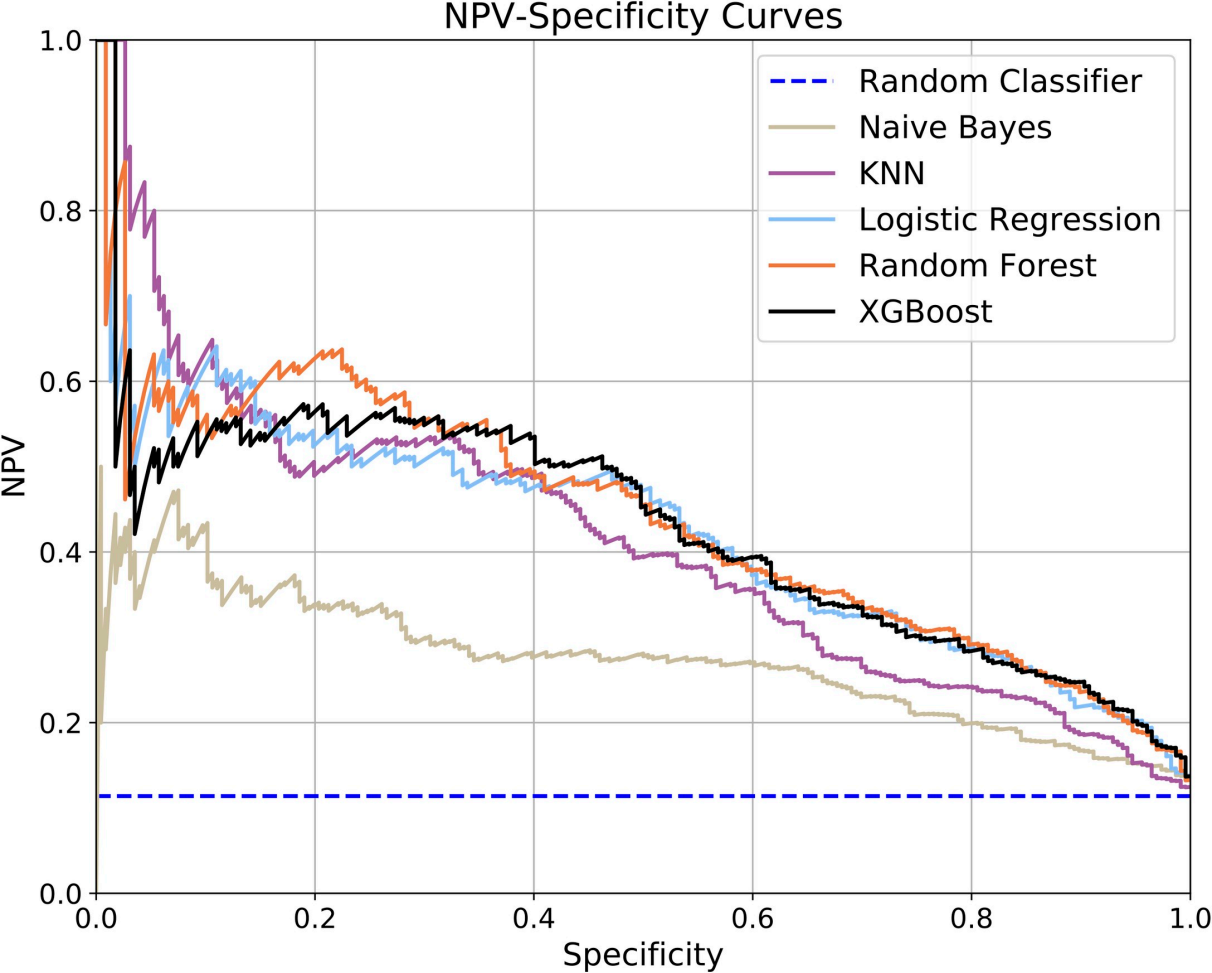
NPV-specificity curves. The curves correspond to the validation fold that yielded the median ANSA.

**Fig 5:**
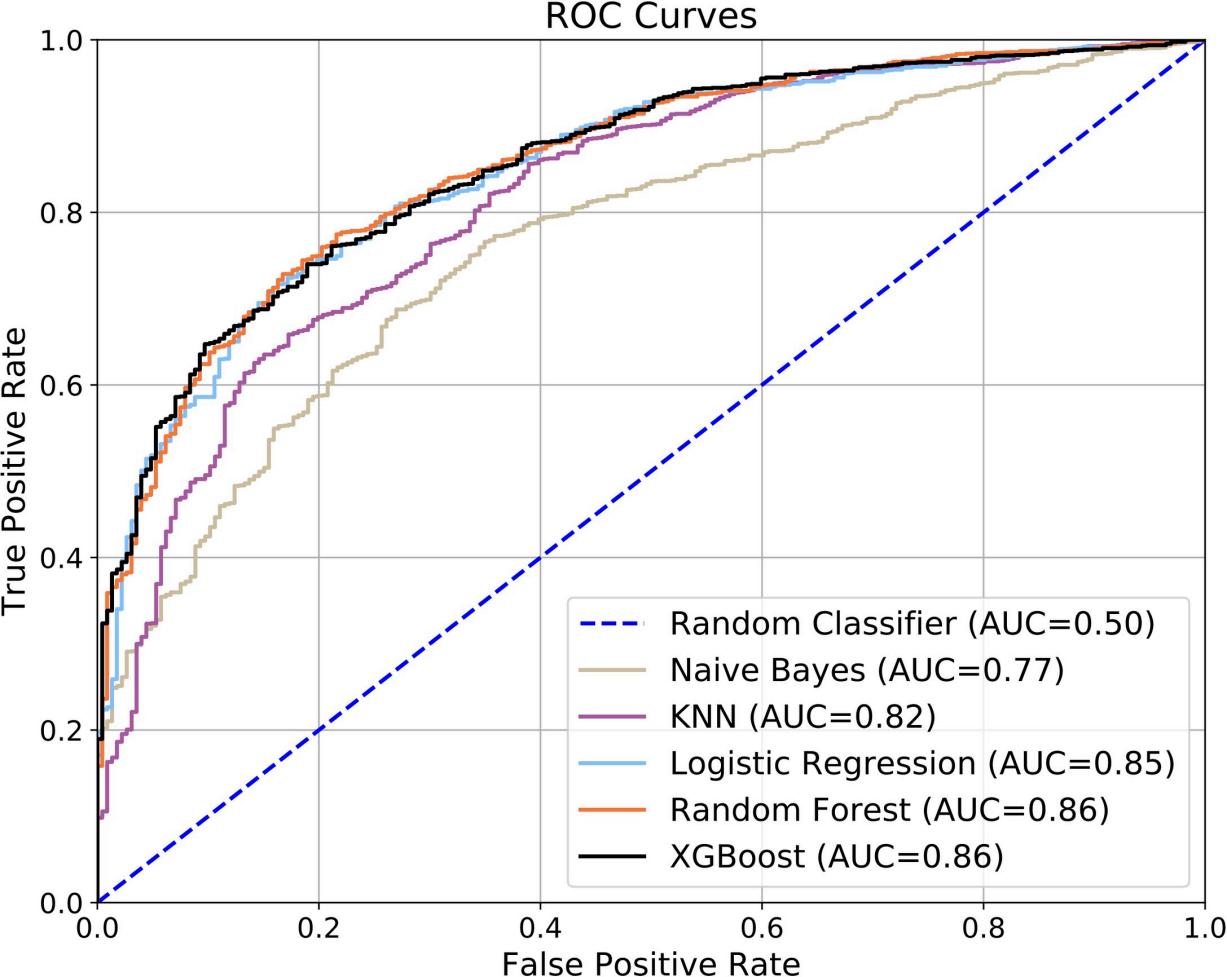
ROC curves. The curves correspond to the validation fold that yielded the median ANSA.

We trained and evaluated five machine learning classifier models to predict future asthma diagnosis persistence given patient data up to age 5 years. Representative NPV-Specificity curves (see Fig 4) indicate that each model performed significantly better than random chance, though the Naïve Bayes model had notably poorer performance. Similarly, the mean and median ANSA also indicate that the models performed well (see Table 2) with the exception of Naïve Bayes. Additional performance metrics at a fixed specificity of 70% on the validation folds are reported in Table 2. For completeness, we also present representative ROC curves (see Fig 5).

**Table 2:** Performance metrics.

Algorithm	Mean ANSA	Median ANSA	NPV	Precision	Recall	F1 Score	Accuracy
Naïve Bayes	0.29 [0.25, 0.33]	0.27	0.25 [0.22, 0.29]	**0.95** [0.95, 0.95]	0.72 [0.69, 0.78]	0.82 [0.80, 0.86]	0.72 [0.69, 0.77]
K-Nearest Neighbors	0.40 [0.32, 0.43]	0.41	0.29 [0.26, 0.32]	**0.95** [0.95, 0.95]	0.78 [0.74, 0.81]	0.85 [0.83, 0.87]	0.77 [0.74, 0.80]
Logistic Regression	0.42 [0.35, 0.45]	0.42	0.32 [0.31, 0.33]	**0.95** [0.95, 0.96]	0.81 [0.80, 0.81]	0.87 [0.87, 0.88]	0.80 [0.78, 0.80]
Random Forest	0.42 [0.34, 0.45]	0.44	0.33 [0.30, 0.35]	**0.95** [0.95, 0.96]	**0.82** [0.79, 0.83]	**0.88** [0.86, 0.89]	**0.81** [0.78, 0.82]
XGBoost	**0.43** [0.38, 0.45]	0.43	**0.34** [0.32, 0.35]	**0.95** [0.95, 0.96]	**0.82** [0.81, 0.83]	**0.88** [0.87, 0.89]	**0.81** [0.80, 0.82]

Values in the second and third columns are the mean and median of the evaluation metric (ANSA), respectively across all 10 cross-validation folds. Values in last five columns represent mean evaluation metrics at fixed specificity of 0.7. The probability of asthma persistence threshold was adjusted individually for each model in each cross validation run to achieve 0.7 specificity. Each metric value is computed as the mean over 10 iterations of cross-validation. Values in brackets indicate the range of the values. Values in bold indicate highest performance for the metric in the given column.

The optimal class balance and feature selection methods for each model are presented in S2 Table. The corresponding optimal hyperparameters for each model are summarized in S3 Table. A comparison of model performance between the optimal class-balance routine and no class-balancing is presented in Table 3. If the optimal class balance routine was found to be no class balance, then a comparison with the next best class-balance routine is presented. The feature selection routine is held constant for each comparison. A similar comparison between feature selection methods holding class balance routine fixed is presented in Table 4.

**Table 3:** Comparison of class-balance routines.

Algorithm	Optimal Class-Balance	None/Suboptimal	Difference
Naïve Bayes	None (0.289)	Tomek (0.286)	0.003
K-Nearest Neighbors	Random under sampling (0.396)	None (0.369)	0.027
Logistic Regression	Random under sampling (0.417)	None (0.411)	0.006
Random Forest	Tomek (0.42)	None (0.419)	0.001
XGBoost	Class weight (0.4272)	None (0.4271)	0.0001

Values in parenthesis represent the mean ANSA values over cross-validation folds. The far-right column indicates the difference in mean ANSA reported in the second and third columns.

**Table 4:** Comparison of feature-selection routines.

Algorithm	Optimal Feature-Selection	None/Suboptimal	Difference
Naïve Bayes	Anova-F (0.289)	None (0.265)	0.024
K-Nearest Neighbors	MultiSURF (0.396)	None (0.394)	0.002
Logistic Regression	Chi squared + ReliefF + Embedded feature selection (0.417)	None (0.285)	0.132
Random Forest	Embedded feature selection (0.42)	ReliefF + Embedded feature selection (0.419)	0.001
XGBoost	Embedded feature selection (0.427)	ReliefF + Embedded feature selection (0.426)	0.001

Values in parenthesis represent the mean ANSA values. The far-right column indicates the decrease in mean ANSA reported in the second and third columns.

The null hypothesis that all of the machine learning models in this study have the same ANSA distributions over the 10 CV folds was rejected based on the Friedman Rank Sum test with p < 0.001. Post-hoc analysis using the exact permutation test (see S4 Table) indicated that all five models had a higher ANSA compared to a random classifier (p < 0.001) and Naïve Bayes had a lower ANSA compared to the other four models (p < 0.001). Additionally, there was some evidence that XGBoost performed better than K-Nearest Neighbors (p < 0.05).

**Fig 6:**
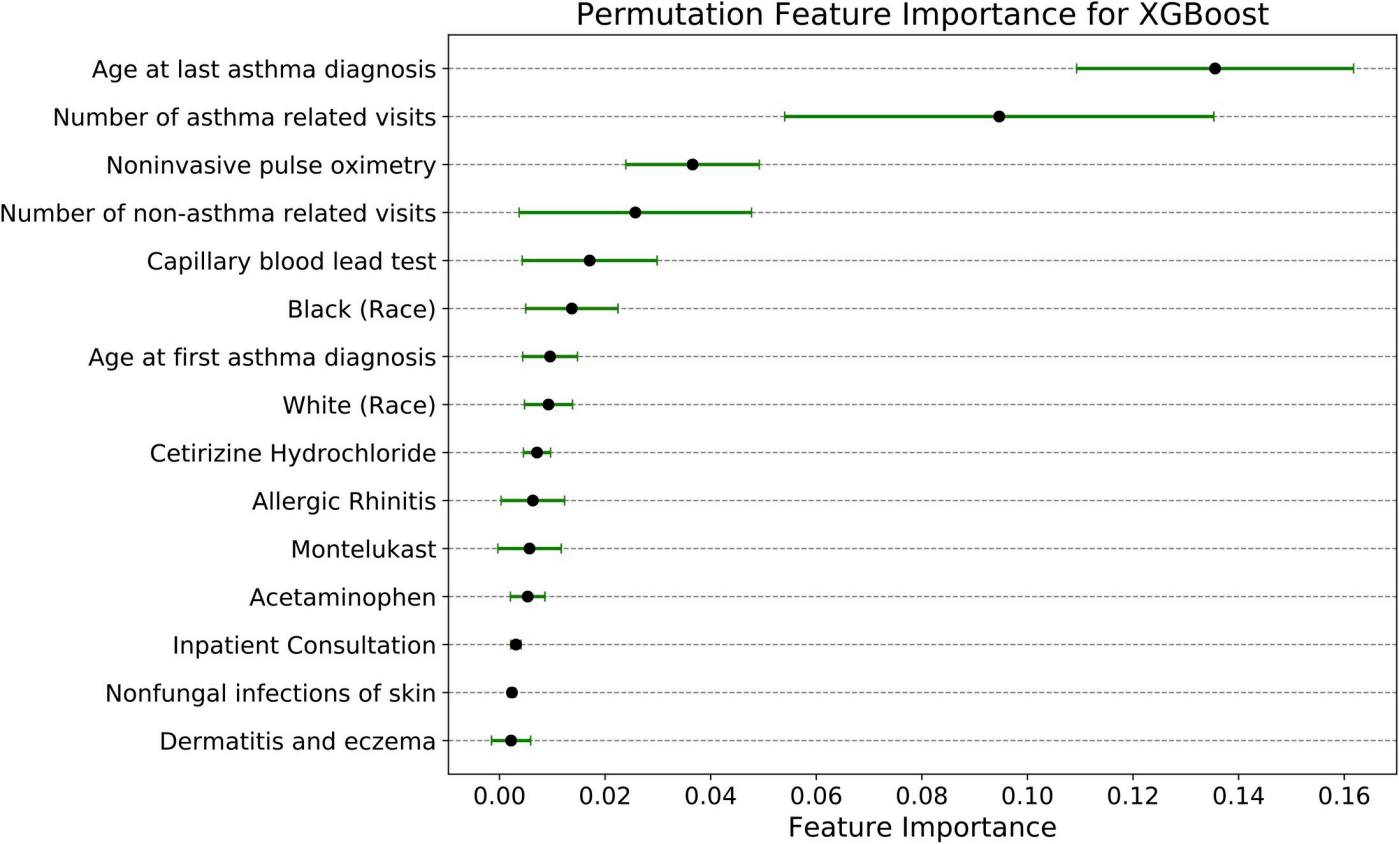
Permutation feature importance scores. The feature importance on the x-axis represents the mean decrease in model performance measured by ANSA when a feature is removed.

Finally, we attempted to gain insight into the model behavior for XGBoost, which was one of our most complex and most accurate models. We performed a permutation feature importance analysis [33] based on the validation set that yielded the median ANSA over the CV folds. The top 15 features in descending order of their importance are summarized in Fig 6.

## 4. Discussion

We developed machine learning models that effectively predict persistent and transient asthma diagnoses. Of the five algorithms considered, XGBoost, random forest and logistic regression performed the best with no statistically significant pairwise differences in ANSA, however the Naïve Bayes classifier had demonstrably lower performance. This is likely because the algorithm makes conditional independence assumptions between the features that may not hold in our dataset. The K-Nearest Neighbor (KNN) algorithm performed better than Naïve Bayes but not as well as the other algorithms. This may result from the high dimensionality of the feature space. The KNN algorithm classifies instances based on a majority voting scheme of nearest neighbors. It has been shown that as dimensionality increases distance between neighbors approaches a constant which degrades nearest neighbor algorithms [34]. Additionally, we examined learning curves (not shown) for XGBoost to evaluate the presence of model over-fitting or under-fitting. This analysis indicated that model over-fitting was not present suggesting that additional training samples using the current feature set will not improve model performance. However, this analysis did indicate the presence of bias which may exist because of insufficient model capacity or because the current feature set does not fully delineate persistent and transient asthma. Since XGBoost is capable of modeling very complex class boundaries, we believe that the likely explanation for the observed bias is inadequate feature parameterization.

Despite having good predictive performance, complex machine learning models like XGBoost often face criticism for their black-box nature. We attempted to alleviate some of these concerns through a permutation analysis to examine feature importance. The permutation feature scores (see Fig 6) revealed that the XGBoost model strongly utilized clinically relevant features. The model picked up on age of last asthma diagnosis under 5 years and total number of asthma-related visits as the most important features. Other features that the model identified, such as self-identified black race and diagnoses of allergic rhinitis, have been demonstrated as asthma risk factors in many studies [35], while others may reflect increased use of the health system for non-asthma respiratory conditions or other reasons (capillary blood lead testing, number of non-asthma visits, acetaminophen use).

The NPV-Specificity curves (see Fig 4) indicate that model performance can vary with the choice of decision threshold which can be tuned to either increase the NPV or the specificity. Arbitrarily decreasing the threshold decreases the number of false negatives but at the same time increases the number of false positives. This could lead to unnecessary treatment for children who would not end up experiencing persistent symptoms, the potential risk of side-effects, and limitations in child and caregiver quality of life. On the other hand, arbitrarily increasing the threshold decreases the number of false positives but increases the number of false negatives. This could lead to children who are at risk of experiencing persistent asthma missing the right treatments resulting in preventable morbidity and even death. Further, in the absence of the model, given an early diagnosis of asthma, long-term and short-term prognoses are equally likely resulting in a sub-optimal outcome. Thus, for the model to really be useful, it is important to find the right threshold and strike a balance between the accepted number of false positives and false negatives. When we considered model performance at a high specificity of 70%, the best performing models were able to identify the positive cases at least 81% of the time with 95% precision.

Although it was difficult to compare our quantitative findings (e.g. ANSA and AUC values) due to the lack of existing literature, our qualitative findings compared favorably with the 2007 birth cohort study [11] by To T. et. al. Consistent with our findings (see Fig 6), the authors of that paper found age of diagnosis and health service utilization to be statistically significant risk factors of persistent asthma. To T. et. al. also found that socioeconomic status was not statistically associated with asthma persistence which was corroborated by our finding of Medicaid enrollment (used as a proxy for socioeconomic status) not being a significant risk factor. However, we did find capillary blood lead testing as an important predictor. Since the test is more routinely performed and recommended in neighborhoods with older housing and higher prevalence of positive tests, the prescription of such a test could be a marker of socioeconomic disadvantage. However, establishing such an association warrants further research. Interestingly, in contrast to their study, we did not find patient sex as an important determinant of asthma persistence. We also found that prescription of the asthma controller medication, Montelukast, prior to the age of 5 was an important feature to the prediction model. Inhaled corticosteroids were simultaneously not found as important. We suspect this is an artifact of representing medications by their ATC codes. Specifically, in our data, all Montelukast variants are represented by a single ATC code, whereas inhaled corticosteroids (e.g. Fluticasone) are represented by several ATC codes which likely diluted their impact.

When dealing with an imbalanced dataset like ours, class balancing techniques have been quite popular in the machine learning research community. Nevertheless, there has been contrasting evidence about their efficacy. While some studies indicate balancing a dataset improves performance, others show that classifiers induced from imbalanced datasets have comparable performance [36]. We tested many well-established under sampling techniques and their combinations. We observed that the efficacy of class balance routines varied with the choice of the ML model (see S2 Table). However, improvements in the model performance by using class balance techniques when compared with no class balancing was very small (statistical test not performed; See Table 3). It is unclear if this is a consequence of our particular classification task and dataset, or a more general result and is a question we will investigate in future research.

When dealing with datasets with a large feature space, feature selection is typically used to eliminate noisy features and avoid model overfitting. We tested many filter methods based on univariate statistical tests as described in Section 2.3. However, univariate statistical tests by their very nature are incapable of handling feature interactions. In contrast, relief based algorithms (RBAs) such as the ReliefF algorithm and its derivatives like the MultiSURF algorithm are capable of detecting feature dependencies [37]. However, these RBAs are nearest neighbor based methods and their performance suffers in large feature spaces. We proposed a novel way to counter this limitation by combining the statistical tests with RBAs. Performing a preliminary feature selection using a statistical test before subjecting the dataset to an RBA based feature selection routine reduces the feature dimension encountered by the RBA thereby potentially improving its efficacy. Similar to class balancing, we found that the choice of the optimal feature selection routine varied with the choice of the ML algorithm (see Tables 4 and S2). Interestingly, filter methods had little impact on the performance of the tree-based models that implicitly use embedded feature selection.

There are some important limitations to our models. Notably, our patients were concentrated in the North-Eastern region of the United States. As a result, the exact models developed in this study may not be generalizable to other geographic regions. However, the model development pipeline outlined in the study is fully reproduceable and can serve as a blueprint for retraining the model on new data. Moreover, we note that the machine learning models developed in this study can only identify correlations, and not causations in the dataset. Although identifying causal pathways may be clinically more relevant, such an analysis was out of the scope of this study. We do think however, that augmenting a dataset like ours with allergen sensitization, genetic, and environmental data and supplementing the ML models with causal analyses can support generation of new knowledge. This is a frontier that we wish to pursue in future research.

## 5. Conclusion

Our results demonstrate that machine learning models can be trained on EHR data to effectively distinguish between persistent and transient asthma cases. Specifically, XGBoost, a tree-based model, was found to be one of the best performing models in this study. It was also found that the model was reliant on clinically relevant features to make predictions; partially addressing model interpretability concerns. However, we note that before the model is deployed as a clinical decision support tool, further research is warranted to test and potentially improve model generalizability by adding other input features as described earlier and evaluating the model on external datasets. We also think that further work on studying the models’ interpretability will play an important role in translating them into clinical practice.

## Supporting information

S1 Fig Pseudo code for the model training procedure. The outer loop picks the class balance and feature selection technique for the cross-validation procedure. The inner loop performs cross-validation for a given choice of hyperparameters and the middle loop scans the hyperparameter space using Bayesian optimization.
(TIFF)

S1 Table Hyperparameter search space. The search space is used as an input by the SMBO algorithm. Details about the hyperparameters can be found in the *scikit-learn* (https://scikit-learn.org/stable/modules/classes.html) and *XGBoost* (https://xgboost.readthedocs.io) documentations.
(DOCX)

S2 Table Optimal pre-processing routines. The class balance and feature selection methods that yielded the highest mean ANSA score over the 10 cross validation folds, for each machine learning model are presented. The far-right column indicates the average number of features selected across the CV folds. Values in brackets represent the range.
(DOCX)

S3 Table Optimal hyperparameters. Hyperparameters that yielded the highest mean ANSA score over the 10 cross validation folds, for each machine learning model are presented. Detailed definitions of the hyperparameters can be found in the *scikit-learn* (https://scikit-learn.org/stable/modules/classes.html) and *XGBoost* (https://xgboost.readthedocs.io) documentations.
(DOCX)

S4 Table Pairwise model performance comparison. Any symbol ⊗ at location (i,j) implies the relationship i⊗j, i.e. > in (i,j) position indicates model in row i performed significantly (p<0.05) better than model in column j as indicated by the exact permutation test. Common acronyms for the algorithms have been used as follows: XGB–XGBoost, RF–random forest, LR–logistic regression, KNN–K-nearest neighbor, NB–naïve Bayes, RC–random classifier.
(DOCX)

S1 File Asthma related medication. CSV file containing asthma-related medication names used to define asthma persistence.
(XLSX)

S2 File Lab measurements. CSV file containing lab measurement names deemed plausibly predictive of persistent asthma diagnosis by our clinician collaborators.
(XLSX)

S3 File Procedures. CSV file containing clinical procedures used as features for model training for which at least 5% of the study cohort had the procedure at least once prior to age 5 years.
(XLSX)
