# Supplementary figures and images for: Personalized prediction of early childhood asthma persistence: A machine learning approach
Source: PLoS One. 2021 Mar 1;16(3):e0247784. doi: 10.1371/journal.pone.0247784 (PMC7920380; doi:10.1371/journal.pone.0247784)

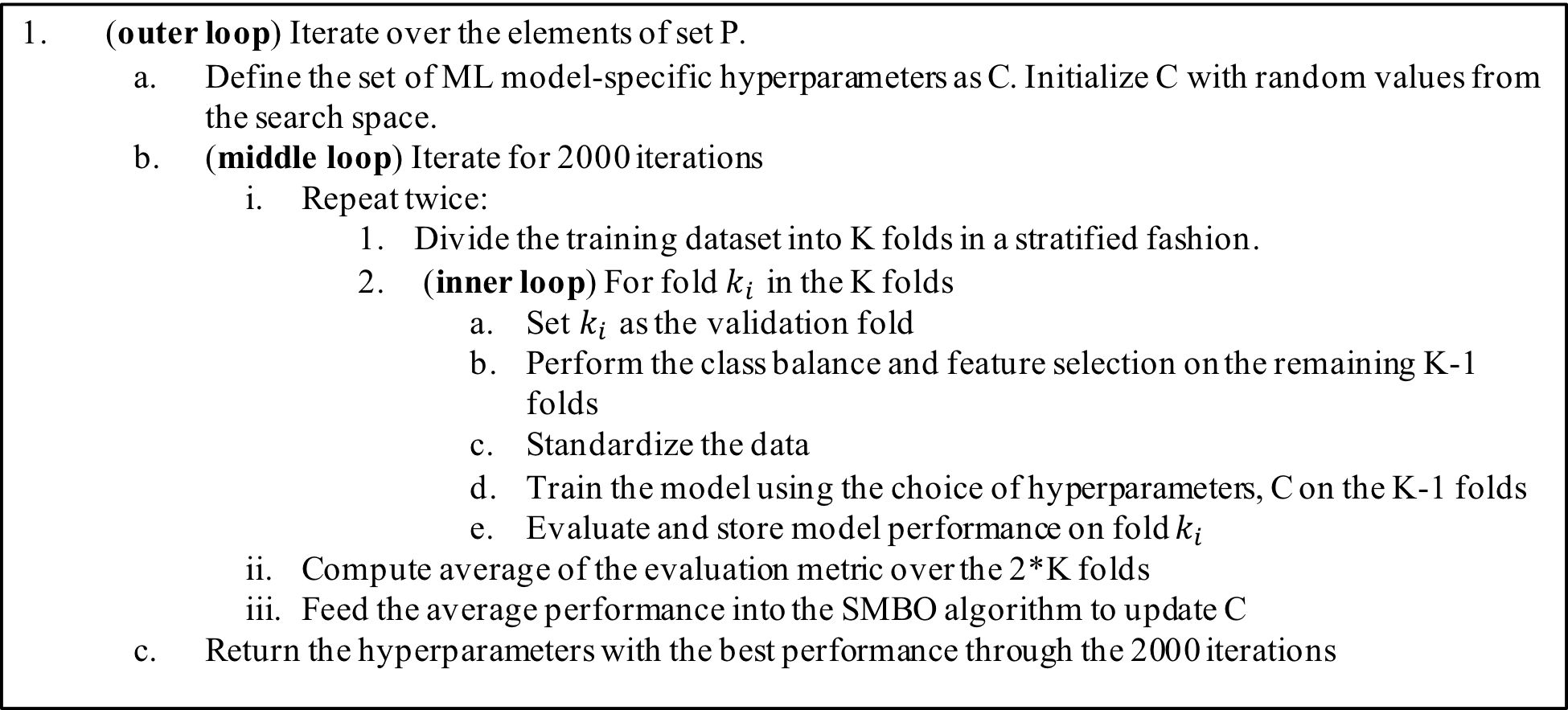

Supplement: S1 Fig — The outer loop picks the class balance and feature selection technique for the cross-validation procedure. The inner loop performs cross-validation for a given choice of hyperparameters and the middle loop scans the hyperparameter space using Bayesian optimization. (TIFF) [file pone.0247784.s001.tiff]
