# Supplementary material for: Personalized prediction of early childhood asthma persistence: A machine learning approach
Source: PLoS One. 2021 Mar 1;16(3):e0247784. doi: 10.1371/journal.pone.0247784 (PMC7920380; doi:10.1371/journal.pone.0247784)
Supplement: S1 Table — The search space is used as an input by the SMBO algorithm. Details about the hyperparameters can be found in the scikit-learn (https://scikit-learn.org/stable/modules/classes.html) and XGBoost (https://xgboost.readthedocs.io) documentations. (DOCX) [file pone.0247784.s002.docx]

S1 Table. Hyperparameter search space. The search space is used as an input by the SMBO algorithm. Details about the hyperparameters can be found in the *scikit-learn* (<https://scikit-learn.org/stable/modules/classes.html>) and *XGBoost* (<https://xgboost.readthedocs.io>) documentations.

| **Algorithm** | **Parameters** | **Values** |
| --- | --- | --- |
| Naïve Bayes | (Distribution, Additive smoothing parameter) | (Bernaulli, uniform[0,2]*), (Multinomial, uniform[0,2]*), (Gaussian, None) |
| Logistic Regression | Penalty | L1, L2** |
|  | Inverse regularization | loguniform[-7,5] † |
| K-Nearest Neighbors | Number of neighbors | range[1, 300] ‡ |
|  | Neighbor weights | uniform, distance ¶ |
|  | Power parameter | 1, 2, 3, 4, 5 |
| Random Forest | Number of trees | range[2, 2000] ‡ |
|  | Minimum leaf node size | uniform[0.01,0.5]* U range[1,1000] ‡ |
|  | Maximum number of features | sqrt, log2 §, None, 0.1, 0.2, 0.4, 0.6, 0.8, 1.0, 2, 5, 10 |
|  | Split criterion | gini, entropy† † |
|  | Bootstrap | True, False |
| XGBoost | Boosting learning rate | uniform[0.01, 0.5]* |
|  | Maximum tree depth | range[1, 100] ‡ |
|  | Number of trees | range[2, 2000] ‡ |
|  | Minimum instance weight needed in child | range[1, 100] ‡ |
|  | Minimum loss reduction required to partition leaf | uniform[0.1, 1]* U range[5,100] ‡ |
|  | Subsample ratio of instance | uniform[0.1,1]* |
|  | Subsample ratio of columns | uniform[0.1,1]* |
|  | L1 regularization coefficient | 0, 1e-5, 1e-2, 0.1, 1, 10, 100 |
|  | L2 regularization coefficient | 0, 1e-5, 1e-2, 0.1, 1, 10,100 |
| *uniform[a,b] indicates parameter values are drawn from a uniform distribution over the interval [a,b]  **L1 and L2 indicate the L1 and L2 norms, respectively  †loguniform[a,b] indicates parameter values are drawn from a log uniform distribution with support a ≤ x ≤ b  ‡ range[a,b] indicates parameter values are drawn uniformly from a discrete distribution in Z over the interval [a,b]  ¶ uniform indicates that all points in the neighborhood of the target point are weighted equally; distance indicates that points in the neighborhood are weighted by the inverse of their distance to the target point  § Sqrt and log2 compute the square root and binary logarithm of the number of features in the dataset, respectively  † †gini refers to the Gini impurity and entropy to the information gain | | |
