# Supplementary material for: Personalized prediction of early childhood asthma persistence: A machine learning approach
Source: PLoS One. 2021 Mar 1;16(3):e0247784. doi: 10.1371/journal.pone.0247784 (PMC7920380; doi:10.1371/journal.pone.0247784)
Supplement: S2 Table — The class balance and feature selection methods that yielded the highest mean ANSA score over the 10 cross validation folds, for each machine learning model are presented. The far-right column indicates the average number of features selected across the CV folds. Values in brackets represent the range. (DOCX) [file pone.0247784.s003.docx]

**S2 Table. Optimal Pre-Processing Routines.** The class balance and feature selection methods that yielded the highest mean ANSA score over the 10 cross validation folds, for each machine learning model are presented. The far-right column indicates the average number of features selected across the CV folds. Values in brackets represent the range.

| **Algorithm** | **Class-Balance** | **Feature Selection** | **Resulting number of input features** |
| --- | --- | --- | --- |
| Naïve Bayes | None | Anova-F | 110.6 [99, 123] |
| K-Nearest Neighbors | Random under sampling | MultiSURF | 426.4 [413, 442] |
| Logistic Regression | Random under sampling | Chi squared followed by ReliefF | 38.4 [33, 43] |
| Random Forest | Tomek | None | 648 |
| XGBoost | Class weight | None | 648 |
