# Supplementary material for: Personalized prediction of early childhood asthma persistence: A machine learning approach
Source: PLoS One. 2021 Mar 1;16(3):e0247784. doi: 10.1371/journal.pone.0247784 (PMC7920380; doi:10.1371/journal.pone.0247784)
Supplement: S3 Table — Hyperparameters that yielded the highest mean ANSA score over the 10 cross validation folds, for each machine learning model are presented. Detailed definitions of the hyperparameters can be found in the scikit-learn (https://scikit-learn.org/stable/modules/classes.html) and XGBoost (https://xgboost.readthedocs.io) documentations. (DOCX) [file pone.0247784.s004.docx]

**S3 Table. Optimal Hyperparameters.** Hyperparameters that yielded the highest mean ANSA score over the 10 cross validation folds, for each machine learning model are presented. Detailed definitions of the hyperparameters can be found in the *scikit-learn* (<https://scikit-learn.org/stable/modules/classes.html>) and *XGBoost* (<https://xgboost.readthedocs.io>) documentations.

| **Algorithm** | **Hyperparameters** | **Values** |
| --- | --- | --- |
| Naïve Bayes | (Distribution, Additive smoothing parameter) | (Multinomial,1.75) |
| Logistic Regression | Penalty | L2 |
|  | Inverse regularization | 0.14 |
| K-Nearest Neighbors | Number of neighbors | 257 |
|  | Neighbor weights | distance |
|  | Power parameter | 2 |
| Random Forest | Number of trees | 1067 |
|  | Minimum leaf node size | 6 |
|  | Maximum number of features | 0.2 |
|  | Split criterion | entropy |
|  | Bootstrap | True |
| XGBoost | Boosting learning rate | 0.49 |
|  | Maximum tree depth | 84 |
|  | Number of trees | 652 |
|  | Minimum instance weight needed in child | 39 |
|  | Minimum loss reduction required to partition leaf | 0.33 |
|  | Subsample ratio of instance | 0.96 |
|  | Subsample ratio of columns | 0.1 |
|  | L1 regularization coefficient | 10 |
|  | L2 regularization coefficient | 100 |
