# Supplementary material for: Personalized prediction of early childhood asthma persistence: A machine learning approach
Source: PLoS One. 2021 Mar 1;16(3):e0247784. doi: 10.1371/journal.pone.0247784 (PMC7920380; doi:10.1371/journal.pone.0247784)
Supplement: S4 Table — Any symbol ⊗ at location (i,j) implies the relationship i⊗j, i.e. > in (i,j) position indicates model in row i performed significantly (p<0.05) better than model in column j as indicated by the exact permutation test. Common acronyms for the algorithms have been used as follows: XGB–XGBoost, RF–random forest, LR–logistic regression, KNN–K-nearest neighbor, NB–naïve Bayes, RC–random classifier. (DOCX) [file pone.0247784.s005.docx]

**S4 Table. Pairwise model performance comparison.** Any symbol ⊗ at location (i,j) implies the relationship i⊗j, i.e. > in (i,j) position indicates model in row i performed significantly (p<0.05) better than model in column j as indicated by the exact permutation test. Common acronyms for the algorithms have been used as follows: XGB – XGBoost, RF – random forest, LR – logistic regression, KNN – K-nearest neighbor, NB – naïve Bayes, RC – random classifier

| Model | XGB | RF | LR | KNN | NB | RC |
| --- | --- | --- | --- | --- | --- | --- |
| XGB |  | ≈ | ≈ | > | > | > |
| RF |  |  | ≈ | ≈ | > | > |
| LR |  |  |  | ≈ | > | > |
| KNN |  |  |  |  | > | > |
| NB |  |  |  |  |  | > |
| RC |  |  |  |  |  |  |
